# Identification of three wheat globulin genes by screening a *Triticum aestivum *BAC genomic library with cDNA from a diabetes-associated globulin

**DOI:** 10.1186/1471-2229-9-93

**Published:** 2009-07-17

**Authors:** Evelin Loit, Charles W Melnyk, Amanda J MacFarlane, Fraser W Scott, Illimar Altosaar

**Affiliations:** 1 Department of Biochemistry, Microbiology and Immunology, Faculty of Medicine, University of Ottawa, Ottawa, Canada; 2 Chronic Disease Program, Ottawa Hospital Research Institute, Ottawa, Canada; 3 Department of Plant Sciences, University of Cambridge, Cambridge, UK; 4 Bureau of Nutritional Sciences, Food Directorate, Health Canada, Ottawa, Canada

## Abstract

**Background:**

Exposure to dietary wheat proteins in genetically susceptible individuals has been associated with increased risk for the development of Type 1 diabetes (T1D). Recently, a wheat protein encoded by cDNA WP5212 has been shown to be antigenic in mice, rats and humans with autoimmune T1D. To investigate the genomic origin of the identified wheat protein cDNA, a hexaploid wheat genomic library from Glenlea cultivar was screened.

**Results:**

Three unique wheat globulin genes, *Glo-3A*, *Glo3-B *and *Glo-3C*, were identified. We describe the genomic structure of these genes and their expression pattern in wheat seeds. The *Glo-3A *gene shared 99% identity with the cDNA of WP5212 at the nucleotide and deduced amino acid level, indicating that we have identified the gene(s) encoding wheat protein WP5212. Southern analysis revealed the presence of multiple copies of *Glo-3*-like sequences in all wheat samples, including hexaploid, tetraploid and diploid species wheat seed. Aleurone and embryo tissue specificity of WP5212 gene expression, suggested by promoter region analysis, which demonstrated an absence of endosperm specific *cis *elements, was confirmed by immunofluorescence microscopy using anti-WP5212 antibodies.

**Conclusion:**

Taken together, the results indicate that a diverse group of globulins exists in wheat, some of which could be associated with the pathogenesis of T1D in some susceptible individuals. These data expand our knowledge of specific wheat globulins and will enable further elucidation of their role in wheat biology and human health.

## Background

Wheat has a primary position in the human diet, and together with maize and rice provides more than 60% of the calories and proteins consumed by the world population [1]. For the majority of the population, ingestion of wheat does not stimulate an immune response. However, in some genetically susceptible individuals, wheat proteins induce an acute mucosal inflammatory response known as celiac disease [2-4] or Baker's asthma [5,6]. Data are also accumulating that dietary wheat proteins promote an inflammatory response in the gut mucosa of patients with autoimmune Type 1 diabetes (T1D) [7-10].

A wheat storage globulin has been associated with the development of T1D [11]. This protein was identified by screening a wheat cDNA expression library with polyclonal antibodies from diabetic BB rats, a model of spontaneous autoimmune T1D [11]. Antibody reactivity to the gene product of one cDNA clone WP5212 was shown to correlate with pancreatic islet damage and inflammation in diabetic BB rats. The WP5212 cDNA shared 90% nucleotide identity with a 1387 nucleotide region of the sequence M81719 which was annotated in the NCBI database as a wheat 7S globulin sequence, and which has subsequently been attributed to barley [12]. It also shares 100% identity with a *Triticum aestivum *assembled transcript (TA61374_4565) designated as a homologue to barley globulin Beg1 precursor.

7S globulin proteins have been previously characterized in barley at the cDNA level and in maize at the protein level [13,14]. A single homologous gene encodes both globulins, Beg1 in barley and Glb1 in maize [13,15]. In wheat, Globulin 1, Triticin and Globulin 2 have been described [16-18]. Other storage proteins have also been shown to be immunomodulatory. Specifically, various vicilins have been identified as major allergens in a variety of foods, including peanut (Ara h 1), cashew (Ana o 1), walnut (Jug r 2), and soybean (Gly m Bd 28K) [19-21].

In an effort to identify the gene(s) encoding WP5212, we screened a wheat Glenlea genomic library using WP5212 cDNA as a probe. This approach enabled us to identify and characterize the three novel wheat genes *Glo-3A, Glo-3B *and *Glo-3C *that encode 7S globulins.

## Results

### Identification of WP5212-like DNA sequences from the hexaploid wheat genome

A total of 25 positive BAC clones were identified by screening 24 high-density filters of the hexaploid wheat *Triticum aestivum *cultivar Glenlea BAC library (3.1× haploid genome coverage) with the WP5212 specific probe. Twenty-two positive clones were confirmed to contain WP5212-like inserts through PCR analysis. Secondary screening by DNA sequencing confirmed three unique sequences.

### Sequencing of WP5212-like sequences from positive BAC clones

Out of 22 candidates, three unique clones, 1333A17, 895N14, and 1324M8 were chosen to be sequenced. Sequencing primers were designed based on conserved regions in cDNAs from WP5212, barley *Beg1 *[GenBank: M64372] and maize *Glb1 *[GenBank: M24845] determined by sequence alignment. Sequencing was conducted by primer walking. Obtained DNA sequences were assembled into contigs of 4406, 3671 and 1457 bp nucleotides for 1333A17, 895N14 and 1324M8, respectively.

### Sequence characterization and open reading frame identification

Contigs from clones 1333A17 and 895N14 contained full genomic sequences for a globulin gene as determined by open reading frame analysis. The globulin gene open reading frame starts at base pair 1213 in BAC clone 1333A17 and at base pair 309 in BAC clone 895N14. For the 1324M8 clone, 1457 bp ORF was delineated, but no stop codon was identified. However, a partial 5' coding sequence was identified, starting at position 109 in 1324M8 contig.

**Figure 1:**
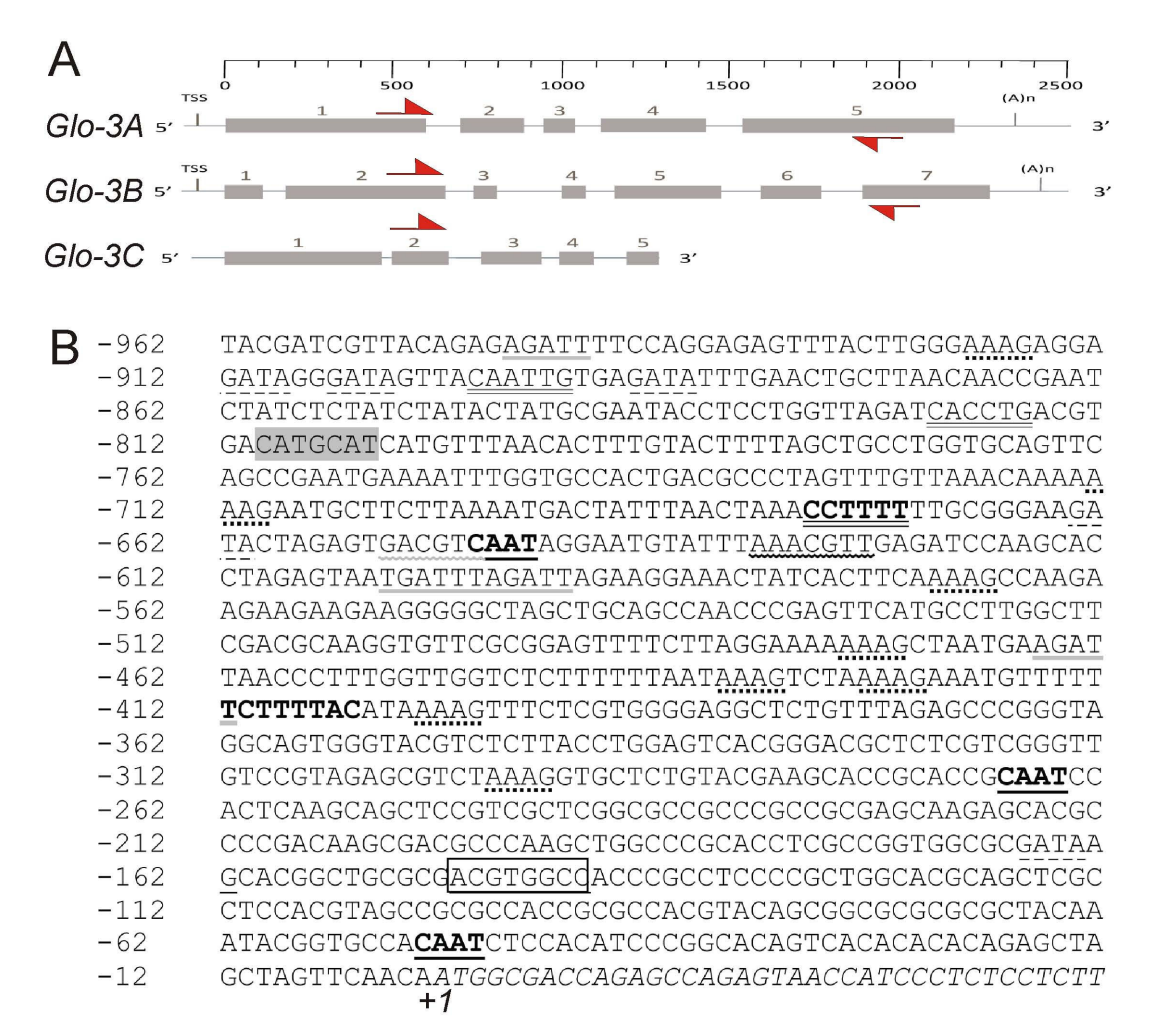
***Glo-3A, Glo-3B and Glo-3C *putative gene structures and promoter elements for *Glo3-A***. (A) The gene structures for *Glo-3A*, *Glo-3B *and *Glo-3C*. Full boxes represent the exons. TSS and (A)n represent the transcriptional start site and polyadenylation sequences, respectively. Red arrows represent RT-PCR primers WPF1 and WPR1. (B) *Glo-3A *promoter region sequence and regulatory elements. Nucleotide positions start one nucleotide upstream of the start of translation at +1 (*ATG*). Putative regulatory elements are indicated: black underlined (CAAT element), boxed (ABRE element), dotted underlined (DOF core recognition sequence), bold neat (prolamine-box), grey underlined (Arr1), bold wave underlined (T-box), grey wave underlined (C-box), bold double underlined (pyrimidine box), dashed underlined (GATA-box), shaded (RY repeat), double underlined (E-box).

No similarity to previously identified wheat globulin genes was found, thus the specific genes were named *Glo-3A*, *Glo-3B *and *Glo-3C *in the order they were identified (originating from BAC clones 1333A17, 895N14, and 1324M8, respectively). All three sequences have been entered into GenBank [GenBank: FJ439135, FJ439136 and FJ439134]. Putative open reading frames, the transcription start sites and the polyadenylation signal sequences were identified (Figure 1A). All sequences contain five to seven exons and four to six introns.

**Figure 2:**
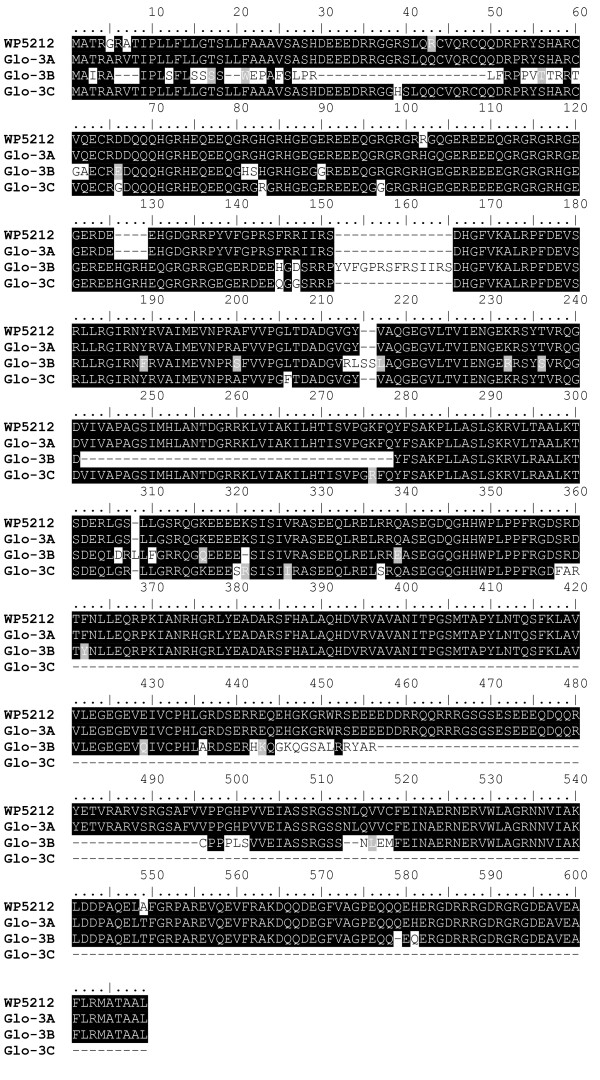
**Alignment of Glo-3 amino acid sequences with WP5212**. Identical amino acids are shaded black. Similar amino acids are shaded grey.

Comparisons between the cDNA clone WP5212 and the coding region of *Glo-3A *showed 99% identity at the nucleotide level. Translated sequence alignment with the predicted amino acid sequence of cDNA clone WP5212 resulted in 99% identity: 583 identical, 3 conserved (G5A, A7V, R102H) and 2 non-conserved (R43Q, A549T) amino acids out of 588 total amino acid residues (Figure 2). Similarly, 99% identity was shared between *Glo-3A *and the assembled wheat transcript [TIGR: TA61374_4565], composed of 230 *T. aestivum *ESTs, known to be 100% identical to WP5212. Importantly, taken together these results demonstrate that the correct terminology for the WP5212 homologue in wheat (previously called Glb1 homologue) should now be named Glo-3A.

The alignment of *Glo-3B *with *Glo-3C *indicated that both genes share 88% and 70% identity at the nucleotide and amino acid level, respectively. *Glo-3B *and *Glo-3C *share 91% and 95% identity with the *Glo-3A *sequence at the nucleotide level, and 74% and 93% identity at the amino acid level. The deduced translation product identified two cupin domains in *Glo-3A *(at 136–260 and 341–508) and one in *Glo-3B *(299–425) and *Glo-3C *(150–264). Cupin domains are common features among vicilins. They are important for nutrition and also play a role in immunoactivation [22,23]. Proteins expressed from *Glo-3A *and *Glo-3B *would be 66.3 kDa and 56.9 kDa, and have predicted pIs of 7.7 and 7.5, respectively.

### Promoter identification and regulatory elements

The 1200 bp of the 5' flanking sequence of *Glo-3A *were analyzed to identify a potential promoter using TSSP, a plant promoter recognition program  and PlantProm database [24]. A putative promoter region was indicated to be between -897 and -43 upstream of the ATG start codon. The promoter region was analyzed for potential *cis*-acting elements using the PLACE database [25]. Multiple putative *cis*-acting elements were identified (Figure 1B and see Additional file 1). The promoter region includes potential binding sites for transcription factors, such as bZIP and DOF. Presence of the abscisic acid response element ABRE suggests the expression of *Glo-3A *is hormone-regulated. Several elements related to tissue specific expression were also found, including E-box, RY repeat, Pyrimidine box, C-box, T-box, and a Prolamin-box.

### Transcriptional activity

The presence of *Glo-3A *mRNA in *T. aestivum *cv. Glenlea seeds 16 days post anthesis demonstrates that this gene is actively transcribed. Primers that correspond to highly conserved regions of *Glo-3A, Glo-3B, Glo-3C*, barley *Beg1 *and maize *Glb1*, so they could bind to a transcript from any of the three sequences, were used to identify Glo-3 transcripts. Since the 3' end of *Glo-3C *was not recovered during primer walking, we designed our primers on the assumption that the 3' region of *Glo-3C *was similar to the other *Glo-3 *genes and, if transcribed, would be amplified during RT-PCR analysis. A cDNA fragment of 900 bp, sequenced from the RT-PCR product, was 100% identical with the *Glo-3A *predicted cDNA sequence in the studied region and corroborated the predicted intron-exon structures (Data not shown). However, RT-PCR products for *Glo-3B *and *Glo-3C *were not observed. Transcriptional activity of *Glo-3A *was further supported by BLAST analysis of wheat EST databases. At least 729 EST sequences from the hexaploid wheat *T. aestivum *shared high similarity with *Glo-3A *and *Glo-3B *and 250 ESTs with *Glo-3C *(see Additional file 2). Among the total of 740 ESTs matching to *Glo-3A*, 722 ESTs were from the developing or mature seed. Relatively few, only 25, ESTs were from *T. aestivum *Glenlea developing seed library 15 DPA, the same cultivar and sampling time used for constructing the genomic library screened in this study and RT-PCR analysis, respectively. With respect to tissue-specific *Glo-3 *gene expression, eight EST sequences from *T. turgidum durum *seedling library were identified. Also, a highly homologous EST clone [GenBank: BQ802077] from *T. monococcum *EST vernalized apex library was found. Mapped ESTs (from Chinese Spring deletion lines) indicated the location of *Glo-3A *to be on the 4AL and/or 4BS chromosome [GrainGenes: BE590748].

### Gene Family Size: determining *Glo-3 *gene copy number in wheat

**Figure 3:**
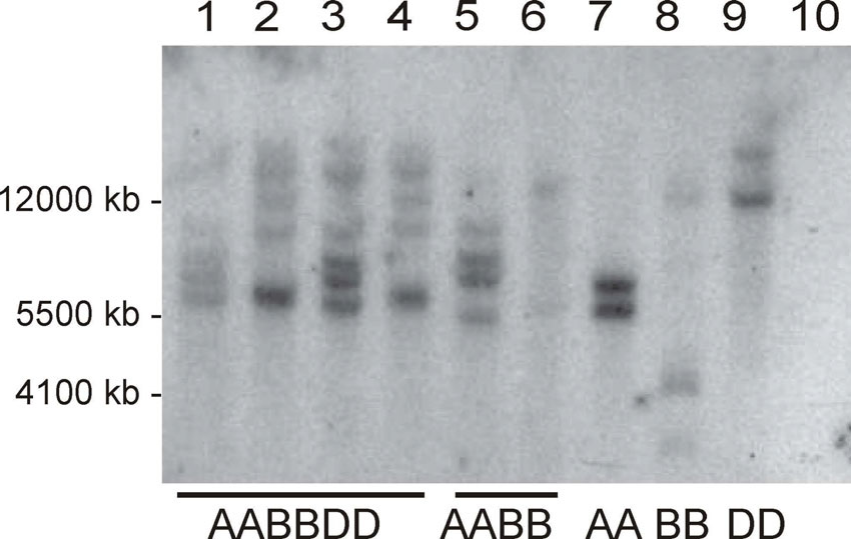
**Southern blot analysis of diploid, tetraploid and hexaploid wheat**. Genomic DNA was digested with Xba I and Xho I and hybridized to *Glo-3*-specific probe.1-*T. aestivum *cv. AC Barrie, 2-*T. aestivum *cv. Glenlea, 3-*T. aestivum *cv. Chinese Spring, 4-*T. aestivum *cv. Spelta, 5-*T. turgidum durum *cv. Kyle, 6-*T. turgidum diccoccum*, 7-*T. monococcum monococcum*, 8-*Aegilops speltoides*, 9-*Ae. tauschii*, 10-*Nicotiana tabaccum*. 1 kb Plus marker sizes are shown in base pairs. Ploidy levels and genomes are indicated underneath the figure.

Wheat cultivars were screened by Southern blot to identify possible genetic lines that might be devoid of WP5212-like proteins. DNA was extracted from cultivars representing all ploidy levels: *T. aestivum *AC Barrie (AABBDD genome), *T. aestivum *Glenlea (AABBDD genome), *T. aestivum *Chinese Spring (AABBDD genome), *T. aestivum *Spelta (AABBDD genome), *T. turgidum durum *Kyle (AABB genome), *T. turgidum dicoccum *(AABB genome), *T. monococcum monococcum *(AA genome), *Aegilops speltoides *(BB genome), and *Ae. tauschii *(DD genome). A 650 bp fragment, amplified from the highly conserved region among all of the three BAC clones was used as a hybridization probe. All of the cultivars examined contained *Glo-3 *genes. Based solely on the number of bands, there are at least two copies of *Glo-3 *genes in A, B and D diploid genomes and at least four homologous copies in tetraploid and hexaploid genomes (Figure 3).

### Immunolocalization

**Figure 4:**
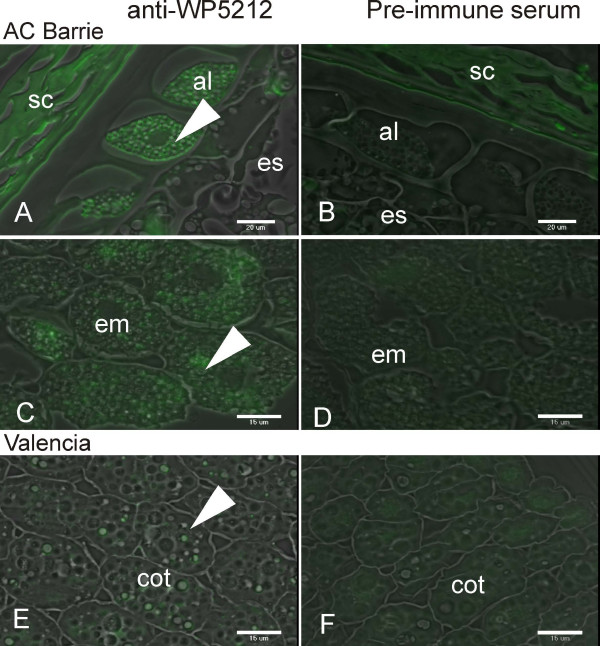
**Immuno-localization of WP5212 in developing wheat grain (cv AC Barrie) and peanut cotyledon (cv Valencia)**. (A) Wheat aleurone (al) cells were positively stained while endosperm (es) remained unstained when sections were stained with WP5212 antibodies. (B) Wheat aleurone layer and endosperm treated with preimmune serum (PIS). Green staining in seed coat (sc) shows unspecific staining. (C) Wheat embryonic (em) tissue stained with WP5212 antibodies. (D) Wheat embryonic cells treated with PIS (E) Peanut cotyledon (cot) stained with WP5212 antibodies. (F) Peanut cotyledon treated with PIS. Green (examples indicated with arrowheads) indicates positive staining.

Immunofluorescence labeling using rabbit anti-WP5212 antibodies revealed the localization of corresponding globulin protein in the aleurone layer and embryo, but not the endosperm of wheat seed sections (Figure 4A, C). Observed fluorescence in wheat seed coat is attributable to background non-specific binding. WP5212 was shown to share immunodominant epitopes to the peanut allergen Ara h1. The same antibodies bound proteins from peanut cotyledon (Figure 4C). WP5212 pre-immune serum did not localize to the embryo, endosperm or aleurone layer in wheat or cotyledons in peanut (Figure 4B, D, F).

## Discussion

We identified three new globulin genes in Glenlea cultivar of hexaploid wheat, *Glo-3A*, *Glo-3B *and *Glo-3C*. These genes share a high degree (73–93%) of nucleotide sequence identity, with occasional amino acid substitutions and indels at specific regions. One of the three, *Glo-3A*, was identified as the genomic sequence corresponding to the WP5212 cDNA sequence [11] sharing 99% identity at the amino acid level. These data confirm that WP5212 is expressed in commercial wheat. The five amino acid difference between WP5212 from AC Barrie and *Glo-3A *from Glenlea could be explained by natural genetic variation due to their origin from two distinct cultivars. The cDNA clone WP5212 was isolated from the AC Barrie cultivar and the *Glo-3A *gene was identified from the Glenlea cultivar. Conservation of storage protein genes in wheat is common. Comparison of puroindoline gene sequences from *Triticum *and *Aegilops *taxa identified an average of 98.4% identity within one taxonomic group [26].

DNA hybridization studies using diploid, tetraploid and hexaploid wheat samples confirmed the presence of *Glo-3*-like genes at all ploidy levels. The restriction enzymes *Xba *I and *Xho *I do not have restriction sites within any globulin sequence. Therefore, these Southern blots are assumed to represent individual copies of *Glo-3 *genes. Multiple bands of different intensities were observed on the fluorograph for all of the studied wheat cultivars (Figure 3). Our results indicate the presence of multiple copies of *Glo-3 *genes in all genomes analyzed (Figure 3). The presence of homologous copies in each ploidy level indicates that a *Glo-3 *sequence has been present during the evolution of all of the wheat lines examined. Additionally, since only one probe designed to recognize one region was used, a broader range of probes may provide a truer measure of *Glo-3 *copy number. The data suggest that the likelihood of identifying or selecting for a *Glo-3 *globulin-negative wheat variety from existing breeding stocks is low. Although BAC screening of the hexaploid wheat Glenlea identified three unique *Glo-3 *genes, it is possible that other homologous sequences exist. There is evidence to support an association between wheat intake and T1D, but the basis of this association and the link to specific molecules remains an open question [7-9]. Nonetheless, the present study using a reverse genetics approach, demonstrates the presence of three novel wheat globulins that are potentially antigenic in patients with T1D and that are present in commercial wheats. Further extensive sequence variance analysis would be required before tools such as siRNA gene silencing could be applied to silence the expression of *Glo-3 *genes.

In polyploid plants, usually only one homologous sequence is transcribed, and the redundant copies are silenced [27]. Of the three genomic globulin copies, RT-PCR analysis showed that the sequence of *Glo-3A *is transcribed 16 days post anthesis (DPA), a time when 7S vicilins are known to be deposited in developing dicot seeds [28-32]. Similarly, *Beg1 *expression has been shown via Northern analysis to start at 15 DPA in barley grain and continues until the maturity of the seed [13]. *Glo-3B *and *Glo-3C *were not found to be expressed in this study at 16 DPA, but further analysis could show their expression at alternate time points.

Screening of GenBank EST libraries with *Glo-3 *genes returned over 700 highly similar ESTs (see Additional file 2). The three datasets exhibit similar results – the same ESTs were identified by all three *Glo-3 *genes, which are attributed to their high degree of identity. The smaller number in the *Glo-3C *dataset is due to the missing 3' end, since ESTs are biased toward the 3'end. Among the others, 25 ESTs from the 15 DPA Glenlea library were found, whereas no matches were found from the 5 DPA library of the same source. This supports the prediction that *Glo-3 *genes in *T. aestivum *Glenlea are expressed 15 DPA. However, screening 22 different libraries (see Additional file 2) identified a small yet significant number of ESTs detected in very early stages of seed development, pre-anthesis flower tissues (5 ESTs) and 7 DPA seeds (6 ESTs). Taken together, the presence of more than 700 corresponding EST sequences in the wheat *Triticum *EST database provides further evidence that *Glo-3 *genes are indeed actively transcribed in a temporally controlled manner.

The majority of the matching ESTs (98%) were isolated from the developing or mature seed. Interestingly, 8 ESTs from *T. turgidum durum *seedling EST library were found to be 99% identical within a 957 bp region, which indicates that *Glo-3 *genes could be expressed in tissues other than seed. To delineate the tightness of spatio-temporal gene regulation requires further experiments to determine the expression patterns of these particular globulins in non-seed tissues of monocots.

Screening the mapped EST database with *Glo-3A *identified a clone previously mapped to the 4AL and/or 4BS chromosome [Genbank: BE590748]. In addition, another EST clone [GenBank: BQ802077] from *T. monococcum*, the A genome progenitor, was found through a GenBank *Triticum *EST database search. These findings suggest that at least one active copy of *Glo-3A *is located on chromosome 4AL/BS. However, the EST databases of *T. monococcum *contain 11,190 ESTs, *Ae. speltoides *4,315 ESTs and *Ae. tauschii *only 116 ESTs, which makes the *in silico *transcriptional analysis of *Glo-3 *genes from different genomes limited or premature.

Putative regulatory elements were identified in the 5' upstream sequence of *Glo-3A *by searching the PLACE database [25] (Figure 1B and see Additional file 1). The prolamin-box located -412 on the negative strand is required for endosperm specific expression, but has been shown to be inactive without a concomitant GCN4 element. This element was not observed in the promoter region of the *Glo-3 *genes supporting our immunolocalization data showing that the WP5212 protein does not localize to the endosperm [33].

The presence of an ABRE element, the ABscisic acid Responsive Element from the early methionine-labeled *Em *gene of wheat [34], at 149 nucleotides 5' to the start codon suggests that *Glo-3A *expression could be regulated by abscisic acid (ABA). Abscisic acid is a hormone that has been shown to regulate maize *Glb1 *expression [35]. Maize Glb1 synthesis and accumulation are positively regulated by ABA through suppressing germination and degradation over the course of embryogenesis [36]. These results suggest that ABA influences storage globulin accumulation by initiating synthesis, suppressing degradation, and inhibiting precocious germination.

E-box and RY elements, also found within the promoter region, could be responsible for embryo-specific expression in wheat as shown in *Arabidopsis *using *phas *promoter [37]. Also, it has been shown that mutations in the RY repeat abolish the seed specific expression [38]. Taken together, the presence of the specific promoter regulatory elements observed in the *Glo-3 *genes suggests not only seed-specific gene expression, but describes an active gene that is specifically expressed in the aleurone layer and the embryo.

The EST analysis also indicates that *Glo-3 *expression is mostly limited to seed tissue (see Additional file 2). Indeed, immunolocalization studies confirmed that expression of *Glo-3 *was restricted to the aleurone layer and embryo within the wheat grain (Figure 4A–D). This is consistent with the observation that the essential endosperm specific regulatory elements, namely the DNA binding site GCN4, are not present in the *Glo-3A *promoter region (Figure 1B). Interestingly, a similar staining pattern was noted in peanut cotyledons (Figure 4E–F). WP5212 was originally shown to share amino acid homology with the peanut allergen Ara h I [11], and our data indicate that antibodies raised against WP5212 also recognize proteins in peanuts. Therefore, we propose that these immunomodulatory proteins could share common antigenic epitopes.

Based on solubility, we speculated that the WP5212 protein could be one of many normal trace contaminants found in wheat gluten and this was demonstrated by 2D gel electrophoresis [11]. Gluten consists of gliadins and glutelins, and these proteins are expressed in the endosperm and are not present in aleurone tissue [39,40]. It is of interest to determine whether WP5212 is present in white flour, which is derived from the endosperm, and in industrial gluten, which is produced from whole wheat.

## Conclusion

WP5212 was first identified by probing a wheat cDNA library with antibodies from diabetic rats. The goal of the current work was to identify WP5212-like genes. Three new globulin gene sequences, *Glo-3A*, *Glo-3B *and *Glo-3C*, from hexaploid wheat were identified and characterized. *Glo-3A *was shown to be the genomic counterpart of the cDNA clone WP5212 and is located on chromosome 4 in the wheat genome. As more full-length sequences become available for the *Glo-3 *genes and proteins, it will be possible to establish their evolutionary relationship. The *Glo-3A *gene was actively transcribed and its protein product localized to the seed coat, aleurone and embryo tissue in the developing seed. We did not identify a *Glo-3*-negative cultivar suggesting that this gene is evolutionarily conserved. These studies have identified the *Glo-3A *gene that shares 99% identity with the cDNA of a WP5212 protein, previously associated with autoimmune T1D and have identified a new globulin gene family that consists of *Glo-3A *and at least two other genes, *Glo-3B *and *Glo-3C*. These findings, identification of the genes whose homologues code for wheat proteins potentially associated with the pathogenesis of T1D, prompt consideration since they are present in the germ and bran layer of commercial wheats, which are significant nutritional components of the human diet.

## Methods

### Plant material and preparation of high molecular weight DNA

Wheat *Triticum aestivum *cv. AC Barrie seeds were provided by Agriculture and Agri-Food Canada, Indian Head Research Farm and Seed Increase Unit (Indian Head, SK). *T. aestivum *cv. Glenlea, *T. aestivum *cv. Chinese Spring, *T. aestivum *cv. Spelta (CDC Bavaria), *T. turgidum durum *cv. Kyle (CN42944), *T. turgidum diccoccum *(CItr 3686), *T. monococcum monococcum*, *Aegilops speltoides *(PI 542261), and *Ae. tauschii *(WGRC2375) seeds were obtained from USDA National Small Grains Research Facility (Idaho). Seeds were grown under standard greenhouse conditions, until shoots were 15–20 cm long. Large-scale purification of DNA was performed as described previously [41]. Briefly, leaves were cut and ground immediately in liquid nitrogen. Thirty mL of 65°C extraction buffer (100 mM Tris-HCl (pH 8.0), 50 mM EDTA (pH 8.0), and 500 mM NaCl) was added to 20 mL of frozen tissue in 50 mL falcon tubes and the contents were shaken. Two millilitres of 20% sodium dodecyl sulphate (SDS) was added to each tube and the tubes were shaken vigorously. The samples were incubated at 65°C for 10 min. Ten mL of 5 M potassium acetate was added to each tube, the tubes were shaken vigorously and placed on ice for 20 min. Samples were clarified by centrifugation at 9000 rpm for 20 min at 4°C in a Beckman JA-12 rotor (Beckman Coulter, Fullerton, California) followed by filtration of the supernatants through one layer of sterile Medicom gauze into sterile 50 mL tubes. RNAse A (10 mg/mL stock) was added at a concentration of 2 μg/mL sample. After ethanol precipitation, the DNA was removed from each tube using a glass Pasteur pipette. The DNA was washed once in 70% ethanol, dried, and dissolved in TE buffer (10 mM Tris-HCl (pH 8.0) – 1 mM EDTA (pH 8.0)) at a concentration of approximately 5–10 mg/mL.

### Restriction digests and Southern blotting

DNA samples were digested with restriction enzymes, *Xba *I and *Xho *I in buffer supplied by the manufacturer (Invitrogen). Gels were alkaline blotted onto Hybond N+ (Amersham, Piscataway, N.J.) membranes using a standard capillary transfer setup with glass plates for support.

### Hybridization studies – BAC library screen and Southern blots

The Glenlea BAC library, kindly supplied by Dr. Cloutier from AAFC-Winnipeg, contains 656,640 clones with an estimated 3.1× haploid genome coverage and has been gridded onto 24 high-density filters [42]. A 459 bp probe (amplified using primers 5'AAAAAGCAGGCTTTCGACGAAGTGTCCAGG 3'and 5'AGAAAGCTGGGTTGCCCAAGAGACTACCCA 3') for the BAC library screening and 650 bp probe (amplified using primers Glb09F and Glb09R) for the Southern blot, consisting of the partial coding regions of WP5212 cDNA clone [11] was labeled with [α-^32^P]dCTP using Ready-to-Go DNA labeling beads (Amersham, Baie d'Urfé, QC) following the manufacturer's instructions and used to screen the filters and membranes. Hybridization was performed as described by Nilmalgoda and co-workers [42]. In short, hybridization buffer was prepared as described by Church and Gilbert (1984), but without BSA. The blots were pre-hybridized overnight at 65°C. The probe was denatured by 10 min boiling, and added to the hybridization tubes. Hybridization was carried out overnight at 65°C. Filters were washed at 65°C in increasingly stringent buffers (2 × sodium chloride/sodium citrate SSC, 0.1% SDS to 0.1× SSC, 0.1% SDS) until counts were approximately 1000 cpm. The following PCR conditions were used for probe preparation: 1× PCR buffer (Invitrogen), 1.5 mmol MgCl_2_/L (Invitrogen), 0.2 mmol/L each of the four dNTPs (Invitrogen), 15 pmol each forward and reverse primers, 1.2 U *Taq *polymerase. The amplification reaction: 94°C for 3 min, followed by 35 cycles at 94°C for 30 s, 60°C for 30 s, and 72°C for 30 s, and a final extension at 72°C for 5 min before cooling to 4°C. The PCR products were purified using the QIAEX II gel extraction kit (Qiagen, Mississauga, ON) according to the manufacturer's instructions.

### RT-PCR

RT-PCR was performed following Invitrogen's protocol. In short, total RNA was isolated from ground seeds (100 mg aliquots) of *T. aestivum *cv. Glenlea, collected 16 days post anthesis. RNA was extracted and purified using RNeasy plant mini kit (Qiagen, Mississauga, ON) according to the manufacturer's instructions. The first strand cDNA was synthesized using the First-Strand Synthesis System for RT-PCR from Invitrogen according to the manufacturer's protocol. The RT-PCR product was amplified with WPF1 and WPR1 primer pair (design based on three *Glo-3 *sequences [GenBank: FJ439134-FJ439136], barley *Beg1 *[GenBank: M64372] and maize *Glb1 *[GenBank: M24845] (see Additional file 3). Expected RT-PCR product sizes on mRNA were 1177 bp and 949 bp for *Glo-3A *and *Glo-3B*, respectively. The same primer pair would amplify PCR products of 1562 bp for *Glo-3A *and 1604 bp of *Glo-3B *on a DNA template. RT-PCR products were cloned into a plasmid vector pGEM-T Easy (Promega, Madison, WI), and their nucleotide sequences were determined using WPR1, WPR2, WPF1, Glb1R, Glb02F and Glb08F primers (see Additional file 3).

### Sequencing

All sequencing was performed at the Ottawa Hospital Research Institute, using Applied Biosystems 3730 DNA Analyzer. Primers used for sequencing are shown in Additional file 3. The sequences identified in this paper have been deposited to GenBank [GenBank: FJ439134; FJ439135; FJ439136].

### Sequence analysis

The contig sequences were analyzed using the NCBI ORF finder  and FGENESH 3.0 alpha  to determine the predicted protein sequence. The conceptually translated proteins were aligned using ClustalX and BioEdit programs [43]. Regulatory elements from the promoter region were identified using the PLACE database [25]. BLASTn analyses were performed using NCBI databases [44], Plant Transcript Assembly database , and wheat mapped EST database (; all URLs last viewed 22.03.09). EST abundance for each Glo-3 mRNA was measured using MegaBlast against GenBank "non-mouse and non-human EST database limited to *Triticum *(total of 1,106,281 sequences, 22.03.09). EST sequences with minimum identity of 86% and minimum E value of 1e-07 were chosen. Presence of the conserved domains was screened with NCBI CDD database [45].

### WP5212 polyclonal serum production

Antigenic WP5212 peptides were predicted by Sigma-Genosys (The Woodlands, TX) technical services (proprietary software). Two WP5212 specific peptides were synthesized for the purpose of polyclonal WP5212 antisera production (Sigma-Genosys). Peptide 1, a 77% pure 16 residue peptide (CRDTFNLLEQRPKIAN), was conjugated to the carrier protein Keyhole-limpet hemocyanin for immunization. Peptide 2 was a 51% pure 15 residue peptide (RGDEAVEAFLRMATA). The purity was determined by mass spectral and HPLC analyses performed by Sigma-Genosys.

The polyclonal antibody production was performed by Sigma-Genosys. Pre-immune serum was collected from two rabbits, after which they were each co-immunized with 200 μg of peptides 1 and 2 in Complete Freund's Adjuvant (day 0). The rabbits were co-immunized with 100 μg of peptides 1 and 2 in Incomplete Freund's Adjuvant on days 14, 28, 42, 56 and 70. Production bleeds from both rabbits were collected on day 40, 63 and 77. WP5212-specific polyclonal antibody production was assessed by 1D SDS-PAGE and Western blotting of recombinant WP5212 isolated from baculovirus infected insect cell lysate (data not shown). Production bleeds were compared to pre-immune serum samples to confirm antibody production.

### LR White sectioning

All fixation and embedding of seeds was performed at 4°C. For London Resin White (LR White) sectioning: mature seeds were fixed in a 4% paraformaldehyde, 0.1 M potassium phosphate buffer (pH 7) for two days. Seeds were placed in two changes of 0.1 M phosphate buffer pH 7 for two hours each, and cut with a razor blade into very thin (2 mm) slices that were placed directly in 70% (v/v) ethanol for one day. Seeds were placed sequentially in 85% ethanol, 100% ethanol and a mixture of 20, 40, 60, 80% (v/v) ethanol: unpolymerized Medium LR White (Sigma-Aldrich, Oakville, ON) for a minimum of two hours per change. Seeds were moved to 100% unpolymerized LR White, and three changes of resin were performed, with a minimum of 12 hours between each change. Finally, seeds were moved to 0.5 ml Beem (Fisher Scientific, Ottawa, ON) capsules containing unpolymerized LR White, and cast at 60°C in a dry oven for four days to polymerize the resin. Polymerized LR White containing seeds were mounted and sectioned with a LKB Ultramicrotome. 2 μm thick sections were placed on Fisher Superfrost Plus slides (Fisher Scientific, Ottawa, ON), and heated on a hot plate for one minute to adhere the sections to the slide.

### Immunofluorescent staining

Mounted sections of wheat AC Barrie and peanut Valencia seeds were washed three times for 10 minutes each in PBS. For primary and secondary immunostaining, the slides were washed three times for 10 minutes each in PBS. Sections were incubated with the primary antibody for one hour at room temperature in a humidity chamber. A 1:1000 (wheat embryo and peanut cotyledon) or 1:5000 (wheat aleurone and endosperm) dilution of the WP5212 antibody was incubated with the sections. After the primary antibody incubation, slides were rinsed three times for 10 minutes each in PBS. 20 μl of the 1:400 dilution of anti-rabbit conjugated Alexa Fluor antibody in PBS was pipetted onto each slide and incubated at room temperature for one hour. Slides were rinsed three times for 10 minutes each in PBS and dried. A drop of Prolong^® ^Gold antifade reagent (Invitrogen, Burlington, ON) was added to each section, 0.2 mm cover slips (Fisher Scientific, Ottawa, ON) were overlayed, and slides were dried overnight at room temperature in the dark. Slides were stored at -20°C. Fluorescent and phase contrast images were visualized using a Zeiss Axiophot microscope (Oberkochen, Germany) equipped with an Olympus DP70 CCD camera. Imaging software, MetaMorph (MetaMorph Imaging Systems, Sunnyvale, California), was used to capture images.

## Abbreviations

(T1D): Type 1 diabetes; (*globulin-3*): *Glo-3*; (EST): expressed sequence tag; (DPA): days post anthesis.

## Authors' contributions

EL performed all experiments (with the exception of immunostaining), analyzed data, contributed all figures and tables except figure 4, and drafted the first manuscript. CWM performed immunostaining of wheat seeds (figure 4). AJM provided the WP5212 antibodies and edited the manuscript. FWS participated in the study design and edited the manuscript. IA designed the study, contributed to data analysis, and edited the manuscript. All authors read and approved the final manuscript.

## Supplementary Material

Additional file 1 **Putative *cis *elements present in the promoter sequence of *Glo-3A***. A table listing all of the *cis *elements present within *Glo-3A *promoter sequence. Click here for file

Additional file 2 **Summary of ESTs resulting from *Triticum *BLAST analysis**. A table providing information about all of the available wheat EST sequences that have similarities to *Glo-3 *sequence. Click here for file

Additional file 3 ***Glo-3 *gene specific primers designed using consensus sequences from *Glo-3A*, *Glo-3B*, *Glo-3C*, full length *Beg1 *(barley), maize *Glb1 *(M24845) and cDNA clone WP5212**. The list of all of the primers used for sequencing. Click here for file
