# Supplementary material for: Identification of three wheat globulin genes by screening a Triticum aestivum BAC genomic library with cDNA from a diabetes-associated globulin
Source: BMC Plant Biol. 2009 Jul 17;9:93. doi: 10.1186/1471-2229-9-93 (PMC2729749; doi:10.1186/1471-2229-9-93)
Supplement: Additional file 1 — Putative cis elements present in the promoter sequence of Glo-3A. A table listing all of the cis elements present within Glo-3A promoter sequence. [file 1471-2229-9-93-S1.doc]

**Additional file 1.** Putative *cis* elements present in the promoter sequence of *Glo-3A.*

| Regulatory element | Consensus sequence | Position | Proposed function | Ref. |
| --- | --- | --- | --- | --- |
| CAAT-box | CAAT | 51, -268, -648 | Seed specificity, storage proteins regulation | [46] |
| ABRE element | ACGTSSSC | - 149 | ABA responsive element* | [34] |
| Dof core recognition sequence | AAAG | frequent | Tissue specific gene expression | [47] |
| Prolamin-box | TGHAAARK | -412 | Endosperm specificity | [47] |
| Arr1 | NGATT | -466, -597, -603, -946 | Response regulator | [48] |
| T-box | AACGTT | -653 | Seed specificity | [49] |
| C-box | GACGTC | -632 | Seed specificity | [49] |
| Pyrimidine box | CCTTTT | - 679 | Seed specificity, GA regulation* | [50, 51] |
| GATA-box | GATA | frequent | Tissue specific gene expression, seed | [52] |
| RY repeat | CATGCAY | -810 | Seed specificity, storage proteins regulation | [37, 53] |
| E-box | CANNTG | -898 | Tissue specific gene expression | [37] |

* ABA - abscisic acid; GA - gibberellic acid.
