# Supplementary material for: Identification of three wheat globulin genes by screening a Triticum aestivum BAC genomic library with cDNA from a diabetes-associated globulin
Source: BMC Plant Biol. 2009 Jul 17;9:93. doi: 10.1186/1471-2229-9-93 (PMC2729749; doi:10.1186/1471-2229-9-93)
Supplement: Additional file 2 — Summary of ESTs resulting from Triticum BLAST analysis. A table providing information about all of the available wheat EST sequences that have similarities to Glo-3 sequence. [file 1471-2229-9-93-S2.doc]

| **Library name** | **Plant/cultivar** | **Tissue** | **Developmental stage** | **Submitter** | **Nr. of ESTs similar to Glo** | | |
| --- | --- | --- | --- | --- | --- | --- | --- |
|  |  |  |  |  | **Glo-3A** | **Glo-3B** | **Glo-3C** |
| Wheat dormant embryo cDNA | *T. aestivum*, Brevor | embryo | mature | O. Anderson, USA | 28 | 28 | 14 |
| wem1c | *T. aestivum* | embryo | 14 DPA | DuPont, USA | 7 | 7 | 5 |
| l:125 | *T. aestivum*, Mercia | embryo | 30 DPA | G. Barker, UK | 115 | 113 | 71 |
| Wheat endosperm cDNA | *T. aestivum*, Cheyenne | endosperm | 5-35 DPA | O. Anderson, USA | 2 | 2 | 0 |
| G550 | *T. aestivum* | grain | 550 degrees/DAP | Genoplante, France | 120 | 122 | 46 |
| wdk2c | *T. aestivum* | kernel | 7 DPA | DuPont, USA | 6 | 2 | 4 |
| wdk3c | *T. aestivum* | kernel | 14 DPA | DuPont, USA | 5 | 3 | 3 |
| wdk4c | *T. aestivum*, Spring wheat | kernel | 21 DPA | DuPont, USA | 3 | 2 | 2 |
| wdk5c | *T. aestivum*, Spring wheat | kernel | 30 DPA | DuPont, USA | 7 | 6 | 5 |
| wkm2n | *T. aestivum*, Spring wheat | kernel | malted 175 h/4 C | DuPont, USA | 2 | 1 | 0 |
| Y. Ogihara unpublished cDNA Wh_EMC | *T. aestivum*, kitaKEI1354 | seed | dormant | Y. Ogihara, Japan | 318 | 311 | 38 |
| TaE15 | *T. aestivum*, Glenlea | seed | 15 DPA | S. Cloutier, Canada | 25 | 25 | 8 |
| TaE05 | *T. aestivum*, Glenlea | seed | 5 DPA | S. Cloutier, Canada | 0 | 0 | 0 |
| Wheat pre-anthesis spike cDNA | *T. aestivum*, Spring wheat | spike | before anthesis | O. Anderson, USA | 5 | 5 | 0 |
| Wheat 5-15 DAP spike cDNA | *T. aestivum*, Chinese Spring | spike | 5-15 DPA | O. Anderson, USA | 3 | 3 | 2 |
| Wheat heat stressed spike cDNA | *T. aestivum*, Chinese Spring | spike | 5-20 DPA | O. Anderson, USA | 9 | 9 | 2 |
| *Triticum aestivum* FGAS: 5 GATE 7 | *T. aestivum*, Norstar | spike | various | P. Gulick, Canada | 9 | 9 | 4 |
| Wheat 20-45 DAP spike cDNA | *T. aestivum*, Chinese Spring | spike and seed | 20-45 DPA | O. Anderson, USA | 15 | 15 | 8 |
| Wheat developing grains cDNA | *T. aestivum*, Butte 86 | whole grain | 3-44 DPA | O. Anderson, USA | 42 | 42 | 33 |
| ITEC WWS Wheat scutellum | *T. aestivum* | scutellum | n/a | W. Schuch, UK | 4 | 3 | 4 |
| *T. turgidum durum* whole plant cDNA | *Triticum turgidum*, Langdon | all | various | O. Anderson, USA | 2 | 1 | 2 |
| *T. turgidum durum* etiol. seedling 20 day | *T. turgidum durum* | seedling | 20 days | R.A. Cifarelly, Italy | 8 | 7 | 5 |
| *T. monococcum* vernalized apex cDNA | *Triticum monococcum* | vernalized apex | 30 days | O. Anderson, USA | 1 | 1 | 1 |
| ITEC SUN Wheat cDNA | *T. aestivum* | n/a | n/a | M. Shariflou, Australia | 4 | 4 | 0 |
| **Total number of ESTs aligned with *Glo* sequences** | |  |  |  | **740** | **705** | **245** |

**Additional file 2.** Summary of ESTs resulting from *Triticum* BLAST analysis.

DPA - days past anthesis; DAP - days after pollination; n/a - not available.
