# Supplementary material for: Identification of three wheat globulin genes by screening a Triticum aestivum BAC genomic library with cDNA from a diabetes-associated globulin
Source: BMC Plant Biol. 2009 Jul 17;9:93. doi: 10.1186/1471-2229-9-93 (PMC2729749; doi:10.1186/1471-2229-9-93)
Supplement: Additional file 3 — Glo-3 gene specific primers designed using consensus sequences from Glo-3A, Glo-3B, Glo-3C, full length Beg1 (barley), maize Glb1 (M24845) and cDNA clone WP5212. The list of all of the primers used for sequencing. [file 1471-2229-9-93-S3.doc]

**Additional file 3.** *Glo-3* gene specific primers designed using consensus sequences from *Glo-3A*, *Glo-3B*, *Glo-3C,* full length barley *Beg1*, maize *Glb1* and cDNA clone WP5212.

| Primer | Primer sequence | Tm (^o^C) |
| --- | --- | --- |
| Glb01F | 5' CACCGTCAAGGAAGGCGATG 3' | 64 |
| Glb01R | 5' ATGGCGGTTGGCGATCTTTG 3' | 62 |
| Glb02F | 5' CCGCGACACCTTCAACCTTC 3' | 64 |
| Glb02R | 5' GCATACCCTCTCGTTCCTCTC 3' | 62 |
| Glb03F | 5' AGAGGAACGAGAGGGTATG 3' | 58 |
| Glb03R | 5' GTAGTAGGTCGGTAGGCAG 3' | 60 |
| Glb04F | 5' GGAGGTGCAAGAGGTGTT 3' | 56 |
| Glb06R | 5'ATGGACAGATACCTTGAGAC 3' | 58 |
| Glb07F | 5' TCTATGACCGCACCCTAC 3' | 56 |
| Glb08F | 5' AGCCCGTGCCATTTGAGAG 3' | 60 |
| Glb09F | 5' TCCCGGCACAGTCACACAC 3' | 62 |
| Glb09R | 5' CGGAGGAGCCTGGACACTT 3' | 62 |
| WPR1 | 5' AACACCTCCTGCACCTCC 3' | 58 |
| WPR2 | 5' CTCGATCACCGTCAGCAC 3' | 58 |
| WPF1 | 5' ACCACGGGTTCGTCAAGG 3' | 58 |
